# Gene duplications are extensive and contribute significantly to the toxic proteome of nematocysts isolated from *Acropora digitifera* (Cnidaria: Anthozoa: Scleractinia)

**DOI:** 10.1186/s12864-015-1976-4

**Published:** 2015-10-13

**Authors:** Ranko Gacesa, Ray Chung, Simon R. Dunn, Andrew J. Weston, Adrian Jaimes-Becerra, Antonio C. Marques, André C. Morandini, Daslav Hranueli, Antonio Starcevic, Malcolm Ward, Paul F. Long

**Affiliations:** Institute of Pharmaceutical Science, King’s College London, 150 Stamford Street, London, SE1 9NH UK; Proteomics Facility, Institute of Psychiatry, Psychology & Neuroscience, King’s College London, 16 De Crespigny Park, London, SE5 8AF UK; Coral Reefs Ecosystems Laboratory, School of Biological Sciences, The University of Queensland, St. Lucia, QLD 4072 Australia; Mass Spectrometry Laboratory, UCL School of Pharmacy, 29/39 Brunswick Square, London, WC1N 1AX UK; Departamento de Zoologia, Instituto de Biociências, Universidade de São Paulo, Rua Matao, Trav. 14, 101, 05508-090 São Paulo, SP Brazil; Centro de Biologia Marinha, Universidade de São Paulo, Rodovia Manoel Hypólito do Rego, km. 131,5, 11600-000 São Sebastião, Brazil; Section for Bioinformatics, Department of Biochemical Engineering, Faculty of Food Technology and Biotechnology, University of Zagreb, Pierottijeva 6, 10000 Zagreb, Croatia; Department of Chemistry, King’s College London, Strand, London, WC2R 2LS UK; Brazil Institute, King’s College London, Strand, London, WC2R 2LS UK; Faculdade de Ciências Farmacêuticas, Universidade de São Paulo, Av. Prof. Lineu Prestes, 580, B16, 05508-000 São Paulo, SP Brazil

**Keywords:** Coral, Nematocyst, Venom, Proteome, Evolution, Hidden Markov model (HMM)

## Abstract

**Background:**

Gene duplication followed by adaptive selection is a well-accepted process leading to toxin diversification in venoms. However, emergent genomic, transcriptomic and proteomic evidence now challenges this role to be at best equivocal to other processess . Cnidaria are arguably the most ancient phylum of the extant metazoa that are venomous and such provide a definitive ancestral anchor to examine the evolution of this trait.

**Methods:**

Here we compare predicted toxins from the translated genome of the coral *Acropora digitifera* to putative toxins revealed by proteomic analysis of soluble proteins discharged from nematocysts, to determine the extent to which gene duplications contribute to venom innovation in this reef-building coral species. A new bioinformatics tool called HHCompare was developed to detect potential gene duplications in the genomic data, which is made freely available (https://github.com/rgacesa/HHCompare).

**Results:**

A total of 55 potential toxin encoding genes could be predicted from the *A. digitifera* genome, of which 36 (65 %) had likely arisen by gene duplication as evinced using the HHCompare tool and verified using two standard phylogeny methods. Surprisingly, only 22 % (12/55) of the potential toxin repertoire could be detected following rigorous proteomic analysis, for which only half (6/12) of the toxin proteome could be accounted for as peptides encoded by the gene duplicates. Biological activities of these toxins are dominatedby putative phospholipases and toxic peptidases.

**Conclusions:**

Gene expansions in *A. digitifera* venom are the most extensive yet described in any venomous animal, and gene duplication plays a significant role leading to toxin diversification in this coral species. Since such low numbers of toxins were detected in the proteome, it is unlikely that the venom is evolving rapidly by prey-driven positive natural selection. Rather we contend that the venom has a defensive role deterring predation or harm from interspecific competition and overgrowth by fouling organisms. Factors influencing translation of toxin encoding genes perhaps warrants more profound experimental consideration.

**Electronic supplementary material:**

The online version of this article (doi:10.1186/s12864-015-1976-4) contains supplementary material, which is available to authorized users.

## Background

Venoms are usually complex mixtures of peptides and proteins colloquially known as toxins. These toxins can disrupt cellular functions or physiological processes, but venoms differ from poisons in that the venom must be delivered through specialised anatomical structures, such as fangs or stinging devices, that inflict a wound to the target prey or predator. This generally accepted definition includes also that toxins are biosynthesised and the venom is then secreted from specialised glands [1]. However, this definition falls short for a group of venomous invertebrates called the cnidarians that do not have any glandular tissues for toxin secretion. Instead, venom is produced by the Golgi apparatus of specialised cells called cnidocysts that are organised for toxin delivery by discharge of a secretory organelle called the cnida, which is unique to cnidarians and a defining characteristic of this phylum [2, 3].

Cnidaria has two major linages; the Anthozoa (sea anemones and corals) and Medusozoa, comprising the classes Staurozoa (stalked jellyfish), Cubozoa (box jellyfish), Scyphozoa (‘true’ jellyfish) and Hydrozoa (*Hydra* and relatives including several species of small jellyfish); see [2, 4] for a recent review. Human envenomation by cnidarians is common and, although seldom life-threatening, fatal contact with certain jellyfish such as the cubozoan *Chironex fleckeri* (the Australian Sea Wasp) is well documented in both the scientific literature and lay press [5]. There have been numerous studies characterising the venoms of many animals, but until recently the toxin component and function of cnidarian venoms was poorly studied and near completely unknown [6]. Still now, patterns for cnidarian venoms are variable and uncertain. We have previously used a high throughput proteomics approach to characterise putative toxins from the nematocysts (a type of cnida) of the coral *Stylophora pistillata* [7] and the hydrozoan jellyfish *Olindias sambaquiensis* [8]. The biological diversity and sequence similarity between these cnidarian toxins and those of completely unrelated higher animals were astounding, suggesting that at least some universal molecular processes leading to toxin diversification might be shared between basal metazoans and diverging lineages of venomous animals.

It is conventionally accepted that venom systems arose by a ‘birth and death’ process following convergent recruitment of ancestral genes that originally encoded non-toxic physiological functions [9]. These genes underwent duplication followed by rapid hyper-mutation independently in different animals to evolve proteins with cytotoxic functions when expressed in venom gland tissues [10, 11]. Adaptive selection has retained useful paralog genes, which in turn has given rise to larger toxin-specific gene families, for example: phospholipase A2, serine proteases, C-type lectins and coagulation factor V, which are regularly present in many venomous animals [12–16]. Venoms diversified additionally as more species-restricted gene families, such as the snake three-finger toxins [17], scorpion cysteine-enriched toxins [18] and the conotoxins of marine cone snails [19], evolved This ‘birth and death’ hypothesis has been refined recently, based upon genome sequence data analysis of predicted proteins from the non-venomous Burmese python *Python molurus bivittatus* [20]. Using tissue specific gene expression profiling, evidence provides that some genes encoding physiological functions are orthologs of toxin encoding genes which are differentially expressed in many different tissue types of the python. Specific recruitment of such orthologs into venom gland tissue followed by ‘birth and death’ evolution would result in paralogs where one copy would ultimately encode a toxic function. This explanation might, therefore, account for the large gene expansions seen in venom gland transcripts of xenophidian snakes [21], as well as that observed in the genome sequence of the highly venomous King Cobra *Ophiophagus hannah* [22]. Reverse recruitment of toxin encoding genes into non-venom gland tissue with reverse conversion of the gene products returning to a non-toxic physiological role has also been predicted from phylogenetic analyses [23] as demonstrated by comparative transcriptome analysis of toxin gene paralogs in venom gland and other tissues of the venomous snake *Bothrops jararaca* (South American pit viper) [24].

Comparative transcriptomics of venomous and non-venomous reptiles however, has cast doubt on the extent to which recruitment and reverse recruitment processes play in the evolution of venom systems [25]. The ‘restriction hypothesis’ confirms previous findings that toxin orthologs are expressed in many tissues of non-venomous reptiles, including salivary glands, suggesting that toxin orthologs have not been recruited but had already existed in glandular tissues [22–24]. Following gene duplication, paralogs can evolve so that expression of one copy, now encoding a toxic function, is restricted to the venom gland, whilst the original copy encoding a non-toxic physiological role remains expressed in other tissues [25]. The extent to which gene duplication has impacted on venom innovation has also been challenged because, although gene duplication in cone snail [26] and snake toxins [27] may occur at an enhanced rate, gene duplication in eukaryotes is generally considered a rare event [28]. In addition, evaluation of transcriptomic dataand sequence analysis of the duck-billed platypus (*Ornithorhynchus anatinus*) genome affirms that gene duplication did not contribute significantly to toxin diversification in this venomous mammal [29].

Other molecular processes that could lead to toxin diversification *in lieu* of gene duplications have been proposed. For example, although experimentally not proven, exon shuffling of primary mRNA transcripts has been suggested as a mechanism to account for active site variation in amino acid sequences of venom gland serine proteases in the snake *Macrovipera schweizeri* (Milos viper) [30]. Likewise, homologous recombination at the DNA or RNA levels may account also for sequence variation in Class P-I and P-II snake venom metalloproteinases (SVMP) in *Bothrops neuwiedi* (Neuweid's lancehead pit viper) [31]. However, such arguments have been based on mapping to sequences outside of known exon splicing sites in cDNA encoding a different SVMP class, which were obtained from the venom transcript of a taxonomically distant snake [32]. Hence, the extent to which toxin diversification can be attributed to processes of gene recruitment and duplication, or indeed recombination and alternative splicing of DNA or RNA, remain largely unexplored. This is principally due to a lack of sequenced genomes of venomous animals from which either true gene duplicates can be identified, or onto which RNA and peptide sequences can be mapped. In direct contrast, post-translational processes including amino acid modifications and protein splicing have been unequivocally established to increase conotoxin diversity in marine cone snail venoms [33].

The sequenced genomes of three cnidarians are currently available; these are *Nematostella vectensis* [34], *Hydra magnipapillata* [35] and *Acropora digitifera* [36]. There are numerous transcriptome libraries also for many cnidarians and, in addition to the nematocyst proteomes we have published [7, 8], the proteome of *H. magnipapillata* has likewise been reported that includes a description of putative toxins [37]. We have made freely available annotation of the predicted proteome of *A. digitifera* at ZoophyteBase (http://bioserv7.bioinfo.pbf.hr/Zoophyte/registration/login.jsp). A search of this database revealed that the predicted toxins of *A. digitifera* are highly homologous to those toxins of many taxonomically distant venomous animals [38]. Having existed since at least the Pre-Cambrian era, Cnidaria are possibly the oldest lineage of extant animals to have evolved means to inject toxins into their prey [4, 39]. If one assumes a single early evolutionary origin of toxin genes, Cnidaria thus provide a unique ancestral anchor to explore the genesis of toxin innovation, which have evolved independently to radiate in other venomous animals [40]. To assess the extent to which gene duplication drives toxin diversification in the Cnidaria, we herein compare the amino acid sequences of *predicted* toxins derived from the translated genome of *A. digitifera* to that of putative toxins *observed* by proteomic analysis of soluble proteins discharged from isolated nematocysts.

## Results

### Identification of potential toxin encoding genes in the *A. digitifera* genome

The translated genome of *A. digitifera* was searched for homology to known animal toxins in the UniProtKB/Swiss-Prot Tox-Prot dataset. The BLAST search used an e-value cut-off selection criterion of 1.0e^−5^ that recovered 950 potential animal toxin homologs. To discriminate potential coral specific toxins from coral proteins with non-toxic physiological functions, these 950 hits were further filtered using an iterative five step process adapted from previously published methods for cnidarian toxin identification [41, 42]. Firstly, only sequences with Reciprocal Blast Best Hit (RBBH) or relaxed RBBH (using the top five BLAST hits for reciprocal BLAST) to sequences in the UniProtKB/Swiss-Prot Tox-Prot dataset with query coverage above 70 % were retained. Secondly, BLASTp comparisons were performed against the entire UniProt database supplemented with additional cnidarian protein sequences [43] and, against a customised database constructed using only cnidarian protein sequences contained within UniProt. Only RBBH or relaxed RBBHs hits were retained having a cut-off e-value of less than 1.0e^−5^ for sequences from both databases . Thirdly, sequences were then manually validated for consistency, and all sequences giving higher scores to non-toxin protein family hits in the cnidarian supplemented UniProt database were discarded. Fourthly, sequences with two or more potential transmembrane domains, or having domain architectures different from known toxins, and Gene Ontology (GO) term assignments unlikely to be related to toxins were also excluded from further examination. Finally, the retained sequences were compared by BLASTp to the translated *A. digitifera* genome, and those with peptide sequences coverage greater than 75 % and e-value homology below 1.0e^−20^ were predicted to be *bona fide* coral specific toxins. A total of 55 potential toxins could be recovered following this five stage filtering process. These 55 potential toxins are shown in Table 1, together with a expectation of likely biological function by inference to a known animal toxin with closest peptide sequence homology. Nearly a quarter (13/55) of the potential *A. digitifera* toxins shared most similar sequence homology to that of other known cnidarians toxins.

**Table 1:** Predicted venom proteome of potential toxins from the translated genome sequence of *Acropora digitifera*

*A. digitifera* protein accession number	Predicted biological effect	Sequence homology (e-value)	Uniprot accession number	Toxin with closest homology	Example of animal species with closest homology
adi_v1.16452	Cytolysin	5.00E-55	J3SBP3	Phosphodiesterase	*Crotalus adamanteus* (Eastern diamondback rattlesnake)
adi_v1.02125	Cytolysin	2.0E-137	J3SBP3	Phosphodiesterase	*Crotalus adamanteus* (Eastern diamondback rattlesnake)
adi_v1.08969	Cytolysin	1.0E-76	F8J2D3	Phospholipase-B	*Drysdalia coronoides* (White-lipped snake)
adi_v1.12172	Cytolysin	4.0E-121	F8J2D3	Phospholipase-B	*Drysdalia coronoides* (White-lipped snake)
adi_v1.14353	Cytolysin	2.0E-83	[43]	Δ-ALTX-Pse	*Phyllodiscus semoni* (Night sea anemone)
adi_v1.09427	Cytolysin	7.0E-93	[43]	Δ-TATX-Avl2a	*Actineria villosa* (Okinawan sea anemone)
adi_v1.16619	Cytolysin	3.00E-88	[43]	Δ-TATX-Avl2a	*Actineria villosa* (Okinawan sea anemone)
adi_v1.16440	Cytolysin	1.00E-12	[43]	Δ-AITX-Aas1a (Bandaporin)	*Anthopleura asiatica* (Giant green sea anemone)
adi_v1.24162	Cytolysin	3.00E-11	P61914	Equinatoxin-2 (Actinoporin)	*Actinia equina* (Beadlet sea anemone)
adi_v1.04835	Cytolysin	2.0E-120	J3SDX8	Lipase	*Crotalus adamanteus* (Eastern diamondback rattlesnake)
adi_v1.23821	Cytolysin	3.00E-101	J3SDX8	Lipase	*Crotalus adamanteus* (Eastern diamondback rattlesnake)
adi_v1.01810	Cytolysin	7.0E-65	J3SDX8	Lipase	*Crotalus adamanteus* (Eastern diamondback rattlesnake)
adi_v1.09322	Cytolysin	4.0E-47	J3RZ81	Lipase	*Crotalus adamanteus* (Eastern diamondback rattlesnake)
adi_v1.00020	Cytolysin	1.0E-39	J3RZ81	Lipase	*Crotalus adamanteus* (Eastern diamondback rattlesnake)
adi_v1.12434	Cytolysin	1.00E-43	A2VBC4	Phospholipase A1	*Polybia paulista* (Neotropical social wasp)
adi_v1.09601	Cytolysin	2.0E-16	Q93109	Equinatoxin-5 (Actinoporin)	*Actinia equina* (Beadlet sea anemone)
adi_v1.10410	Cytolysin	4.0E-52	Reference [43]	Δ-AITX-Ucs1a (Urticinatoxin)	*Urticina crassicornis* (Christmas sea anemone)
adi_v1.12125	Cytolysin	2.00E-26	J3S836	Phosphodiesterase	*Crotalus adamanteus* (Eastern diamondback rattlesnake)
adi_v1.06821	Cytolysin	2.0E-78	A3QVN9	Hyaluronidase	*Bitis arietans* (African puff adder)
adi_v1.16469	Cytolysin	5.00E-13	P81383	L-amino-acid oxidase	*Ophiophagus hannah* (King cobra)
adi_v1.12311	Cytolysin	2.0E-16	A7L035	Toxin CfTX-1	*Chironex fleckeri* (Box jellyfish)
adi_v1.16921	Disrupts haemostasis	3.00E-28	D2X8K2	Phospholipase A2	*Condylactis gigantea* (Giant Caribbean sea anemone)
adi_v1.16374	Disrupts haemostasis	8.0E-32	Q58L94	Prothrombin activator (Notecarin)	*Notechis scutatus scutatus* (Tiger snake)
adi_v1.15821	Disrupts haemostasis	5.0E-41	Q4QXT9	Coagulation factor X	*Tropidechis carinatus* (Rough scaled snake)
adi_v1.08904	Disrupts haemostasis	5.0E-35	Q76B45	Serine-type endopeptidase (Blarina toxin)	*Blarina brevicauda* (Northern short-tailed shrew)
adi_v1.19445	Disrupts haemostasis	8.0E-112	C6JUN2	Metalloprotease (SVMP)	*Philodryas olfersii* (Green snake)
adi_v1.14946	Disrupts haemostasis	1.0E-30	B6EWW8	Snake venom 5’-nucleotidase	*Gloydius brevicaudus* (Korean slamosa snake)
adi_v1.12298	Disrupts haemostasis	4.0E-15	Q4PRC6	Snaclec 7	*Daboia siamensis* (Eastern Russel’s viper)
adi_v1.18989	Disrupts haemostasis	6.00E-08	Q66S13	Fish venom Natterin-4	*Thalassophryne nattereri* (Niquim)
adi_v1.19802	Disrupts haemostasis	1.0E-19	A7X3Z0	C-type lectin (Lectoxin-Thr1)	*Thrasops jacksonii* (Jackson’s black tree snake)
adi_v1.06850	Disrupts haemostasis	9.0E-16	A8QZJ5	Cytotoxin-1	*Millepora dichotoma* (Net fire coral)
adi_v1.22096	Disrupts haemostasis	2.00E-68	D8VNT0	Ryncolin-4	*Cerberus rynchops* (Dog-faced water snake)
adi_v1.19322	Induces immune response	4.0E-19	P35779	Venom allergen 3	*Solenopsis richteri* (Black imported fire ant)
adi_v1.01092	Induces immune response	5.0E-36	J3SFJ3	Translationally-controlled tumor protein homolog	*Crotalus adamanteus* (Eastern diamondback rattlesnake)
adi_v1.11797	Neurotoxin	3.0E-30	P00615	Phospholipase A2	*Oxyuranus scutellatus scutellatus* (Australian taipan)
adi_v1.18628	Neurotoxin	8.0E-15	Q9TWL9	Phospholipase A2 (Conodipine-M)	*Conus magus* (Magician’s cone snail)
adi_v1.05505	Peptidase	1.0E-41	C9WMM5	Serine carboxypeptidase	*Apis mellifera* (Honeybee)
adi_v1.05180	Peptidase	7.0E-52	B2D0J5	Serine carboxypeptidase	*Apis mellifera* (Honeybee)
adi_v1.11218	Proteinase inhibitor	3.0E-17	Q6ITB9	Kunitz-type serine protease inhibitor (Mulgin-3)	*Pseudechis australis* (King brown snake)
adi_v1.23374	Proteinase inhibitor	8.0E-17	P0C8W3	Kunitz-type serine protease inhibitor (Hg1)	*Hadrurus gertschi* (Desert hairy scorpion)
adi_v1.09855	Unknown	3.0E-136	B2DCR8	SE-cephalotoxin	*Sepia esculenta* (Golden cuttlefish)
adi_v1.20368	Venom maturation	2.0E-51	K7Z9Q9	Metalloprotease (Nematocyte expressed protein 6)	*Nematostella vectensis* (Starlet sea anemone)
adi_v1.17845	Venom maturation	3.0E-44	K7Z9Q9	Metalloprotease (Nematocyte expressed protein 6)	*Nematostella vectensis* (Starlet sea anemone)
adi_v1.13648	Venom maturation	4.0E-38	K7Z9Q9	Metalloprotease (Nematocyte expressed protein 6)	*Nematostella vectensis* (Starlet sea anemone)
adi_v1.07444	Venom maturation	1.0E-28	C9D7R3	Metalloprotease (Astacin-like toxin)	*Loxosceles intermedia* (Brown spider)
adi_v1.23604	Venom maturation	3.0E-21	C9D7R2	Metalloprotease (Astacin-like toxin)	*Loxosceles intermedia* (Brown spider)
adi_v1.15751	Venom maturation	7.00E-37	A0FKN6	Metalloprotease (Astacin-like toxin)	*Loxosceles intermedia* (Brown spider)
adi_v1.20292	Venom maturation	5.0E-28	B1A4F7	Venom dipeptidyl peptidase 4	*Vespula vulgaris* (Yellow jacket wasp)
adi_v1.15074	Venom maturation	2.0E-28	B1A4F7	Venom dipeptidyl peptidase 4	*Vespula vulgaris* (Yellow jacket wasp)
adi_v1.09733	Venom maturation	9.0E-29	Q9TXD8	Serine type endopeptidase	*Agelenopsis aperta* (North American funnel-web spider)
adi_v1.03437	Venom maturation	6.0E-13	Q3SB11	Calglandulin	*Tropidechis carinatus* (Australian rough-scaled snake)
adi_v1.01102	Venom maturation	5.0E-38	Q3SB11	Calglandulin	*Tropidechis carinatus* (Australian rough-scaled snake)
adi_v1.05162	Venom maturation	1.0E-47	J3S9D9	Phospholipase A2 activation (Reticulocalbin-2)	*Crotalus adamanteus* (Eastern diamondback rattlesnake)
adi_v1.10508	Venom maturation	5.0E-53	Q8MMH3	Protein-glutamate O-methyltransferase	*Pimpla hypochondriaca* (Parasitoid wasp)

### Identification of potential gene duplicates

**Fig. 1:**
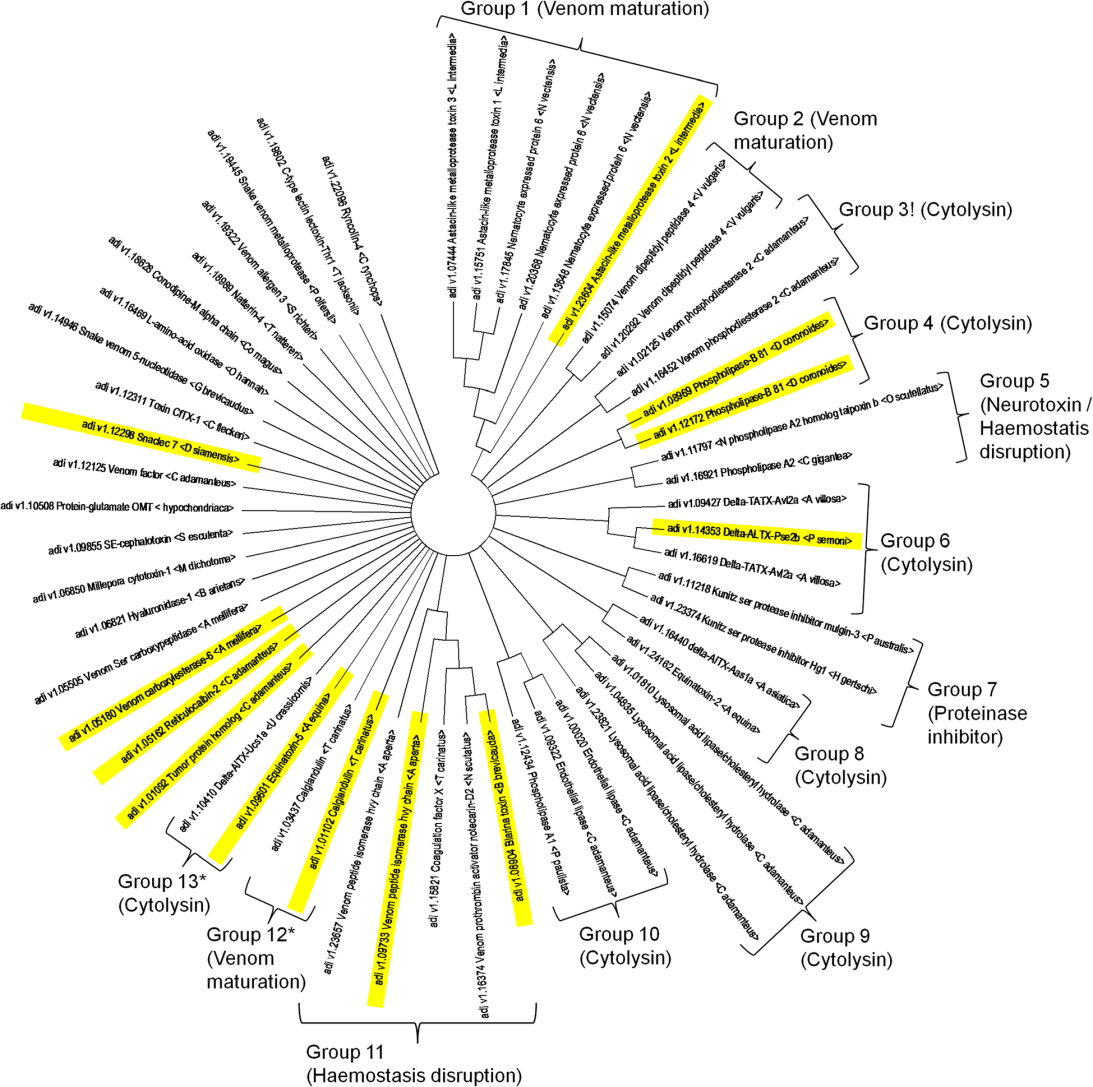
Gene duplication prediction by clustering of *Acropora digitifera* predicted toxins. Clustering was performed using the HHCompare tool and verified by Maximum Likelihood and Maximum Parsimony based methods. Groups marked with * are detected by Maximum Likelihood and Maximum Parsimony based clustering, while groups marked ! were not detected. Proteins highlighted in yellow were detected by high throughput mass spectrometric protein analysis of soluble proteins from discharged nematocysts.

Evaluation of  the role that gene duplication plays in the evolution of toxin diversity requires phylogenetic analysis of sequence data to identify related paralogs from many closely related species. No such data exists for coral species; hence, potential gene duplicates were used as the most likely sequences to be best related to true paralogs. Gene duplicates were identified using a newly developed HMM-HMM based hierarchical clustering tool called HHCompare. Clustering was also performed using standard Maximum Likelihood and Maximum Parsimony phylogenetic methods. All three methods grouped together all of sequences related by identical function (Fig. 1), although there was a slight difference in the number of groups generated by the different methods (Additional file 1). Tajima’s test of neutrality was performed on each group containing more than 2 domain sequences and, in all cases produced a D statistic greater or equal to 4, indicating balancing selection. When taking the results from the three clustering methods together (Fig. 1), the positioning of 36/55 (65 %) sequences within specific groups inferred that these sequences had arisen following gene duplication events. These 36 sequences could be divided amongst 13 groups with predominantly cytotoxic or toxic protease activities. The remaining 19 sequences could not be grouped and were regarded as singlets, again with mainly cytotoxic activities, possibly involved in affecting haemostasis, immune function, neurotoxicity or toxin maturation.

### Identification of potential toxins in the proteome of *A. digitifera* nematocysts

Mass spectral data of peptide fragments obtained from tryptic digests of soluble proteins extracted from discharged nematocysts were first matched for identity to the predicted toxins of *A. digitifera* (Table 1). Stringent identity criteria of two peptide matches at greater than 95 % sequence similarity were selected that gave just 12 homologous matches, representing 22 % (12/55) of the potential toxins in the translated genome sequence. A MASCOT search (i.e., two peptide matches with >95 % sequence similarity) of the spectral data for matches to the predicted proteome of *Symbiodinium* clade B1 was performed to also identify any endosymbiotic algal peptide sequences with homology to predicted *A. digitifera* toxins. No potential contaminating *Symbiodinium* clade B1 proteins were identified despite using a BLAST search with a stringent e-value cut off selection criterion of 1.0e^−20^. The venom toxins of *A. digitifera* had a relatively narrow profile of predicted biological activities such to include phospholipases and pore forming toxins, toxic peptides and peptides predicted to disrupt haemostasis or immune function. Metalloproteases and other peptidases possibly involved in venom toxin maturation were also annotated as part of the expected toxin proteome. Of the 36 peptides attributed to gene duplication, 6 were detected in the proteome which represented 50 % (6/12) of the total peptides in the expressed venom. Manual validation of mass spectra for annotation of 19 potentially unique *A. digitifera* coral toxins was assessed by searching the PRIDE proteomics data repository (http://www.ebi.ac.uk/pride/archive/) for the dataset named ‘Acropora_Digitifera_Toxins’, with sequences in FASTA format are also available from ZoophyteBase (http://bioserv7.bioinfo.pbf.hr/Zoophyte/registration/login.jsp [38]).

## Discussion

Toxin diversification in venoms is traditionally accepted to have arisen by convergent recruitment of genes that have evolved independently within the glandular tissues of diverse animal lineages, following common molecular processes of DNA sequence duplication and deletion [1, 9–11, 20, 24, 25]. Yet, the concept that gene recruitment, sequence duplication and sequence deletion alone are sufficient to explain the surprising chemical diversity of toxins in venoms is increasingly being challenged as genome, transcriptome and proteome data from venomous animals are becoming available [29, 33, 44]. Cnidaria is likely to be the most basal of extant metazoans to be venomous, so we used *Acropora digitifera* for which we had already annotated the predicted proteome [38] to evaluate the extent to which gene duplication could account for toxin diversification in this reef-building coral.

Here, a BLAST homology search of the *A. digitifera* predicted proteome against the UniProtKB/Swiss-Prot Tox-Prot dataset, followed by a stringent five step process to exclude proteins with possible non-toxic physiological functions [41–43], uncovered 55 potential toxins with homology to higher animal toxins (Table 1). This was a low number of potential toxin encoding genes in comparison to that of the two venomous vertebrates for which genome sequences are presently available. Such, there were 107 potential toxin encoding genes identified by similarity to known toxins encoded in the genome of the Duck-billed platypus *Ornithorhynchus anatinus* [29], and 69 predicted toxin encoding genes with homology to toxin families were identified in the genome sequence of the King cobra *Ophiophagus hannah* [22]. However, there was a disparity between the higher numbers of predicted toxin encoding genes that had arisen from likely duplication events identified in this study (36/55, 65 %) as compared to much lower numbers of gene duplicates in the Duck-billed platypus and King cobra genomes. Of the 107 platypus genes with significant sequence similarity to known toxins, only 16 (15 %) were likely to have evolved subsequent to a duplication event; this low number would suggest that the venom of the platypus is diversifying slowly and likely under negative selection. Indeed, the 16 gene duplicates were not members of any major known lethal toxin gene families, and so the venom is unlikely to be under strong adaptive (i.e., positive) evolutionary pressure, thereby producing venom of low potency [29]. This would agree with the likely purpose attributed to platypus venom, which is to incapacitate rather than to kill mating competitors [44], a widespread common sexual selection pattern among mammals. In contrast, the 69 potential toxin encoding genes predicted in the genome of the King cobra have undergone massive expansion, with 30 (i.e., 43 %) likely to have arisen following gene duplication. Of these 30 duplicates, 25 were concentrated in just three major lethal toxin gene families, namely the three-finger toxins, phospholipase A2 and snake venom metalloproteinase enzymes [22]. This high number of gene duplications is consistent with natural selection for specific prey, which requires highly toxic and lethal venom that is evolving quickly to adapt to molecular co-evolution of prey resistance [45].

Evaluation of the role gene duplication plays in the evolution of toxin diversity in basal Metazoa requires bioinformatics methods to identify putative gene paralogs. There are currently two standard approaches based on either comparing the positions of paralogs on phylogenetic tree relationships or by assessing the degree of identity between sequences using BLAST similarity searching methods. Both methods require genomic, transcriptomic or proteomic data obtained from many closely related species in order to identify related paralogs. There are sequenced genomes only for three distantly related cnidarians available in the public domain, and so, tree and BLAST based approaches to identify paralogs is not dependable. Currently available clustering methods such as cd-hit and BLASTClust (ftp://ftp.ncbi.nih.gov/blast/documents/blastclust.html) from the NCBI–BLAST package [46] can be used to infer potential orthology, but do not provide an evolutionary perspective, and such fall short in precision because they use BLAST-like algorithms. Comparison of similarity between groups of potential orthologs based on generating and then comparing hidden Markov models (HMMs) does allow inference of evolutionary distance. However, there are currently no tools available that compare HMMs and then cluster orthologous proteins to allow potential paralogs to be detected within ortholog clusters. For this reason we have developed a new tool called HHCompare. It implements well tested HHsuit programs for HMM generation and HMM vs HMM comparisons [47]. HHCompare then uses iterative pairwise HMM vs HMM comparisons to generate related ortholog groups based on high HMM-HMM similarity (e-value cut-off less than 1.0e^−20^) and then generates relationship trees to cluster the orthologous groups, thereby allowing potential orthologs in and between cluster groups to be detected. In this study, such a low e-value cut-off would only cluster extremely similar orthologous proteins, and so this approach was considered a proxy for likely gene duplication in the absence of sequences from closely related species. The strength of this clustering compared favourably against two standard methods of approach (Additional file 1). The 55 predicted toxins encoded in the *A. digitifera* genome formed 13 clusters with two or more sequences and 19 singlets (Fig. 1). This requires that an astounding 65 % of the predicted venom of *A. digitifera* had likely arisen subsequent to gene duplication, which is far greater than the total expansion of toxin genes reported in the King cobra venom (43 % [22]). This degree of duplication is nearly equivalent to gene expansions reported for specific toxin families in other venomous animals. Conotoxin genes are thought to be the most rapidly evolving in the Metazoa with 70 % of the A-superfamily of conotoxin genes having been established by gene duplication [26]. In sharp contrast, genes encoding the sphingomyelinase D toxin in sicariid spiders are believed to be composed of only 4.4 % of gene duplicates [48]. To our knowledge, *A. digitifera* has the greatest percentage of toxin encoding gene duplications yet reported in the genome of any venomous animal to date.

To assess what adaptive selective pressures might drive and maintain such massive gene expansions in *A. digitifera*, the expressed venom proteome was determined empirically using high throughput mass spectrometric protein analysis. When matched predicted toxins against the translated proteome sequence, and surprisingly only 22 % (12/55) of the predicted proteome could be identified using strict spectral identification parameters. Although peptides likely to be products from gene duplicates accounted for 50 % (6/12) of the toxic proteome, the high number of potential toxins not detected in the venom proteome might reflect poor promotor recognition and therefore weak expression of very recently duplicated genes such that protein abundance is less than the detection limits of the proteomics method [49]. Such a high number of gene duplicates would suggest that the venom is evolving rapidly under adaptive, positive selection. However, with so many of the gene duplicates not seemingly expressed in the empirically determined proteome would, in fact, indicate contrarily that the venom of *A. digitifera* has low toxicity since it is evolving gradually under negative selection. This is in broad agreement with data comparing multiple alignments of amino acid sequences and calculations of amino acid substitution rates, particularly for the sea anemone peptide neurotoxins and pore-forming toxins, which show these cnidarian toxins are under negative selection and thus are highly conserved [50]. Likewise, critical examination of the evolution of three species across cnidarian lineages (the anthozoan sea anemone *Anemonia viridis* (Actinaria), the scyphozoan jellyfish *Aurelia aurita* and the hydrozoan *Hydra magnipapillata*) agrees also with our data that venom of the anthozoan *Acropora digitifera* (Scleractinia) shows little evidence for diversification through positive selection [41].

The putative biological activities of the toxins in both the predicted and observed *A. digitifera* venom were dominated by cytotoxic phospholipases and pore forming toxins (Table 1 and Fig. 1). This is not unusual compared to the known or predicted pharmacological effects of toxins in other cnidarian venoms. For example, in anthozoans, of which sea anemone venoms are the most widely studied in all of the Cnidaria, their venoms are composed mainly of pore forming toxins and peptide neurotoxins [51]. Other anthozoan venoms are less widely studied, but our proteomic analysis of toxins from the coral *Stylophora pistillata* (Scleractinia) predicts that in this coral species venoms are also composed predominantly of cytotoxic peptides and neurotoxins [7]. The venoms of hydrozoans, such as those of the genus *Millepora* (commonly known as ‘fire corals’ and well known for human envenomation causing sever irritation) and *Hydra,* are composed mainly of cytolysins, phospholipase and haemolytic enzymes [52]. *A. digitifera* does feed on microscopic phytoplankton and zooplankton, however, like all of the reef-building corals, *A. digitifera* has evolved an endosymbiotic metabolic partnership with photosynthetic dinoflagellates of the genus *Symbiodinium* (Dinophyceae) which is essential for survival in the nutrient-poor waters of tropical marine environments [53, 54]. The biological relevance of a largely cytolytic toxic arsenal could reflect a possible defensive role to deter fish predation and death by fouling organisms, including attack by coral-excavating sponges (Clionidae) which are strong competitors of corals for space on the reef shelf [55–57]. Biochemical studies to assign specific pore-forming activities to the *A. digitifera* cytolysins will require in future a comprehensive comparative review of pore-forming toxins in Cnidaria to better understand the provenance and biological relevance of these toxins to the life history strategy of these animals [58]. It is a well-accepted concept that toxin gene acquisition follows duplication of genes encoding non-toxic physiological functions [1, 9]. It follows that the toxin encoding genes that were considered as singlets in this study would have most likely have arisen following gene duplication that occurred in the very distant past such that strict evidence for duplication events could not be detected with the methods employed here. Developing an evolutionary clock to determine if the timing of gene duplication events and emergence of specific toxin gene families is correlated with a transition of cnidarians from sessile animals in photo-autotrophic symbiosis to free living heterotrophic lineages is worthy of future research.

## Conclusions

This is the first study to combine genome analysis and proteomics data to critically examine venom innovation in the Cnidaria and the relevance of gene duplication in toxin diversification in particular. After filtering proteins with likely non-toxic physiological function, 55 potentially unique coral toxins have been described. Exploring selection pressures and processes driving the evolution of venom is problematic in Cnidaria since few genomes of related species have been sequenced. Here we exemplify a new bioinformatics tool called HHCompare that overcomes the severity of this impediment. Using this tool, predicted toxin encoding genes of the coral *A. digitifera* could be divided into orthologous groups that are the closest representation to gene duplicates currently possible, which is consistent with groupings determined by conventional phylogenetic methods. Of the 55 toxins, 36 (65 %) are likely established by gene duplication, which represents the largest gene expansion as a percentage proportion of all toxin encoding genes identified in the genome in any venomous animal reported to date. Only 22 % of these peptides were detected in the expressed proteome of discharged nematocysts, suggesting that the venom had evolved for predator defence rather than an offensive role for prey capture. Biochemical validation of toxin activities is now warranted so that full annotation of *A. digitifera* coral specific toxins can be deposited in publically available protein databases. Gene expansion by gene duplication appears crucial to toxin evolution in the basal Metazoa such as exemplified by the Cnidaria. Factors influencing translation of these gene products to enhance venom potency provides a fascinating avenue for further study.

## Methods

### Isolation of nematocysts from coral

Fragments of 3 colonies of the hermatypic coral *A. digitifera* were collected from reef flat sites adjacent to the Heron Island Research Station (S 23° 13’ 30”, E 15° 11‘54”), Great Barrier Reef, Australia in November 2013 and were immediately snap frozen in LN_2_ for transport to the laboratory. The coral fragments were airbrushed on ice with 30 mL of Ca^2+^ free artificial seawater (pH 8.2/32 ppt) for tissue removal. The homogenised tissue slurry (2 mL) was placed on top of a dilution gradient density column consisting of 2 mL each of 30 %, 50 %, 70 % and 90 % (w/v) polyvinyl pyrrolidone (Percoll®: Sigma) in artificial seawater and cooled on ice for 20 min prior to centrifugation at 4 °C for 10 min at 280 × *g*. Following centrifugation, the 50–70 % layer that contained the highest concentration of undischarged nematocysts was collected and then freeze dried (MicroModulo-230 freeze drier in combination with a RVT100 refrigerated vapour trap, Thermo Savant). Corals were collected under permit G12/35434.1 issued by the Great Barrier Reef Marine Park Authority and coral material was transferred to the United Kingdom in accordance with CITES institutional permits AU053 and BG029.

### Proteomics

To extract soluble proteins, 500 μL of extraction buffer containing 50 mM triethylammonium bicarbonate, 0.04 % SDS (w/v), 1 × Complete Mini Protease Inhibitors (Roche) and 1 × Complete Mini Phosphatase Inhibitors reagent (Roche) was added to the freeze-dried coral material. The material was vortex for 1 min and then placed on ice for 1 min; the procedure was repeated 10 times. The material was disrupted with a probe sonicator (model: VC250, Sonics & Materials Inc.) whilst on ice for a total of 15 sec using a duty cycle of 40 % and an output of 3. The material was centrifuged at 13,000 × *g* for 15 min at 4 °C. The protein concentration in the cleared supernatant was then measured by Nanodrop spectroscopy (Thermo Scientific) by averaging the results of three determinations. An extract portion containing 30 μg of soluble protein in 2 × Laemmli buffer (Sigma) was heated for 10 min at 90 °C and loaded onto a 4–12 % (w/v) NuPAGE Novex Bis-Tris gel (Life Technologies) for separation by 1D SDS-PAGE electrophoresis using 2-(*N*-morpholino)-ethanesulfonic acid (MES) buffer alongside Novex SeeBlue Plus2 pre-stained molecular weight standards. Electrophoresis was carried out at 150 V for approximately 100 min. The gel was fixed, Coomassie Blue-stained, de-stained and visualised by scanned image. The entire gel lane was sectioned into 15 equal portions and each section was divided into 2 mm^2^ portioned for in-gel digestion. Briefly, cysteine residues were reduced with 10 mM dithiothrietol and alkylated with 55 mM iodoacetamide in 100 mM ammonium bicarbonate to form stable carbamidomethyl derivatives. Trypsin (Promega) solution was added to the gel sections at 13 ng/μL in 50 mM ammonium bicarbonate and digestion was carried out at 37 °C overnight. The supernatant was retained and the peptides were extracted from the gel sections by two washes with 50 mM ammonium bicarbonate and acetonitrile. Each wash involved shaking for 15 min before collecting the peptide extract and pooling with the initial supernatant. Pooled peptide extracts were then lyophilised. Lyophilised peptides were re-suspended in 30 μL of 50 mM ammonium bicarbonate per gel section prior to LC-MS/MS analysis with 10 μL of each sample injected. Samples were analysed sequentially beginning with the largest molecular weight region on a Thermo Fisher Scientific Orbitrap Velos Pro mass spectrometer coupled to an EASY-nLC II (Thermo Fisher Scientific) nano-liquid chromatography system. Samples were trapped on a 0.1 × 20 mm EASY-Column packed with C18-bonded ultrapure silica, 5 μm (Thermo Fisher Scientific) and separated on a 0.075 × 100 mm EASY-Column packed with C18-bonded ultrapure silica, 3 μm (Thermo Fisher Scientific). Columns were equilibrated in 95 % buffer A (99.9 % deionised water, 0.1 % formic acid) and 5 % buffer B (99.9 % acetonitrile, 0.1 % formic acid). Peptides were resolved over 50 min at a flow rate of 300 nL/min with a gradient of 5–40 % buffer B for 40 min followed by a gradient of 40–80 % buffer B for 5 min and held at 80 % buffer B for a further 5 min. Mass spectra ranging from 400 to 1800 Da (m/z) were acquired in the Orbitrap at a resolution of 30,000 and the 20 most intense ions were subjected to MS/MS by CID fragmentation in the ion trap selecting a threshold of 5000 counts. The isolation width of precursor selection was 2 units and the normalised collision energy for peptides was 35. Automatic gain control settings for FTMS survey scans were 10^6^ counts and FT MS/MS scans were 10^4^ counts. Maximum acquisition time was 500 ms for survey scans and 250 ms for MS/MS scans. Charge-unassigned and +1 charged ions were excluded for MS/MS analysis. Raw MS data were processed for database spectral matching using Proteome Discoverer (Thermo Scientific) software. MASCOT was used as the search algorithm with the variable modifications: carbamidomethylation of cysteine and oxidation of methionine. A digestion enzyme of trypsin was set allowing up to three missed cleavages. A parent ion tolerance of 10 ppm and a fragment ion tolerance of 0.5 Da were used.

### Bioinformatics

The peptide sequences for the approximately 5000 toxins deposited in the UniProtKB/Swiss-Prot Tox-Prot dataset (www.uniprot.org/program/Toxins, [59]) were downloaded in FASTA format. Likewise, the predicted proteomes of *A. digitifera* (http://marinegenomics.oist.jp/genomes/downloads?project_id=3, [36]) and *Symbiodinium* clade B1 (http://marinegenomics.oist.jp/genomes/downloads?project_id=21, [60]) were also downloaded in FASTA format and the three datasets were used as query searches for MS/MS spectra. All dataset search results were reviewed by loading the Mascot result files into Scaffold 4 (www.proteomesoftware.com). BLASTp searches were performed to assess local similarities between sequences in the *A. digitifera* and *Symbiodinium* clade B1 datasets and the UniProtKB/Swiss-Prot Tox-Prot dataset using program version 2.2.27+ from NCBI (ftp://ftp.ncbi.nlm.nih.gov/blast,executables/blast+/2.2.27/, [46]). The outputs from these comparisons were parsed and filtered using a custom assembled program written in Python (www.python.org) to select for high scoring segment pairs with e-values selected with a cut-off value below 1.0e^−5^. Sequences of high scoring segment pairs were filtered to remove proteins with likely physiological functions involving Reciprocal Blast Best Hit (RBBH) analysis [61] domain architecture prediction using InterProScan5 (http://www.ebi.ac.uk/Tools/pfa/iprscan5/), a search of gene ontology terms (http://www.ebi.ac.uk/QuickGO/) and prediction of transmembrane domains using TMHMM Server 2.0 (http://www.cbs.dtu.dk/services/TMHMM/). Grouping of the truncated high scoring segment pairs used our new Hidden Markov Model (HMM) based comparative software designated ‘HMMCompare’ that is assembled in Python. ‘HMMCompare’ is freely available at http://bioserv.pbf.hr/HHCompare-master.zip and is implemented using programs from the HHsuite version 2.0 compiled for the Debian based Linux OS (http://wwwuser.gwdg.de/~compbiol/data/hhsuite/releases/, [47]). Multiple alignments of the truncated sequences were constructed using ClustalW version 2.1 compiled for the Debian based Linux OS (ftp://ftp.ebi.ac.uk/pub/software/clustalw2/2.1/). Phylogenetic clustering was also performed using Maximum Likelihood and Maximum Parsimony methods in MEGA 6.0 [62] with multiple alignments generated using MUSCLE (http://www.ebi.ac.uk/Tools/msa/muscle/). The clusters were tested for neutral evolution using Tajima’s Test of Neutrality [63] implemented in MEGA 6.0.

## Additional file

Additional file 1:
**Clustering and phylogenetic analysis of** ***Acropora digitifera*** **toxins.** HMM based hierarchical clustering (HHCompare): HHCompare clustering was performed at HMM-HMM similarity e-value of 1.0e-20. Following the clustering, sequences within each group were aligned using MUSCLE. For three sequence groups, phylogentic trees were constructed using Minimal Evolution method, while larger groups were analyzed using Maximum Likelihood (ML) method. In case of ML, evolutionary model was inferred by MEGA 6.0 model selection tool, based on Sample-corrected Akaike information criterion (AICc). **Figure S1.** HMM-based hierarchical clustering of coral toxins. Each split indicates HMM-HMM similarity with e-value below 1.0e-20. **A**: Group 1, ML analysis using LG + G model with 4 discrete gamma categories. **B**: Group 6, Minimal Evolution analysis. **C**: Group 9, Minimal Evolution analysis. **D**: Group 10, Minimal Evolution analysis. **E**: Group 11, ML analysis using JTT model with 4 discrete gamma categories. **Figure S2.** Phylogenetic analysis of HMM clustered groups. **Figure S3.** Maximum likelihood based clustering of coral toxins. Sequence names are as following: coral sequence id, followed by evidence for expression (T stands for True and indicates protein was detected in proteomic analysis of nematocyst, while F stands for False and lack of detection). Last part of sequence name is assigned annotation based on Uniprot ToxProt toxins enriched by Anemone toxins. HMM-clustering generated groups are marked on the tree and groups not generated by HMM clustering, but detected by ML clustering are marked by *. **Figure 4.** Maximum parsimony based clustering of coral toxins. (DOCX 61 kb)
