# Supplementary material for: Gene duplications are extensive and contribute significantly to the toxic proteome of nematocysts isolated from Acropora digitifera (Cnidaria: Anthozoa: Scleractinia)
Source: BMC Genomics. 2015 Oct 13;16:774. doi: 10.1186/s12864-015-1976-4 (PMC4604070; doi:10.1186/s12864-015-1976-4)
Supplement: Additional file 1: — Clustering and phylogenetic analysis of Acropora digitifera toxins. HMM based hierarchical clustering (HHCompare): HHCompare clustering was performed at HMM-HMM similarity e-value of 1.0e-20. Following the clustering, sequences within each group were aligned using MUSCLE. For three sequence groups, phylogentic trees were constructed using Minimal Evolution method, while larger groups were analyzed using Maximum Likelihood (ML) method. In case of ML, evolutionary model was inferred by MEGA 6.0 model selection tool, based on Sample-corrected Akaike information criterion (AICc). Figure S1. HMM-based hierarchical clustering of coral toxins. Each split indicates HMM-HMM similarity with e-value below 1.0e-20. A: Group 1, ML analysis using LG + G model with 4 discrete gamma categories. B: Group 6, Minimal Evolution analysis. C: Group 9, Minimal Evolution analysis. D: Group 10, Minimal Evolution analysis. E: Group 11, ML analysis using JTT model with 4 discrete gamma categories. Figure S2. Phylogenetic analysis of HMM clustered groups. Figure S3. Maximum likelihood based clustering of coral toxins. Sequence names are as following: coral sequence id, followed by evidence for expression (T stands for True and indicates protein was detected in proteomic analysis of nematocyst, while F stands for False and lack of detection). Last part of sequence name is assigned annotation based on Uniprot ToxProt toxins enriched by Anemone toxins. HMM-clustering generated groups are marked on the tree and groups not generated by HMM clustering, but detected by ML clustering are marked by *. Figure 4. Maximum parsimony based clustering of coral toxins. (DOCX 61 kb) [file 12864_2015_1976_MOESM1_ESM.docx]

**Supplementary Data File 1: Clustering and phylogenetic analysis of *Acropora digitifera* toxins**

***HMM based hierarchical clustering (HHCompare):***

HHCompare clustering was performed at HMM-HMM similarity e-value of 1.0e-20. Following the clustering, sequences within each group were aligned using MUSCLE. For three sequence groups, phylogentic trees were constructed using Minimal Evolution method, while larger groups were analyzed using Maximum Likelihood (ML) method. In case of ML, evolutionary model was inferred by MEGA 6.0 model selection tool, based on Sample-corrected Akaike information criterion (AICc).

Figure 1. HMM-based hierarchical clustering of coral toxins. Each split indicates HMM-HMM similarity with e-value below 1.0e-20.

Following the HMM-based clustering, phylogenetic trees were constructed for groups with 3 or more sequences.

*A: Group 1, ML analysis using LG+G model with 4 discrete gamma categories*

*B: Group 6, Minimal Evolution analysis*

*C: Group 9, Minimal Evolution analysis*

**

*D: Group 10, Minimal Evolution analysis*

*E: Group 11, ML analysis using JTT model with 4 discrete gamma categories*

Figure 2. Phylogenetic analysis of HMM clustered groups

***Maximium likelihood (ML) clustering:***

ML based clustering was performed using MEGA 6.0. Sequences were aligned using MUSCLE, JTT+Gamma (4 discrete gamma categories) evolutionary model was determined by MEGA 6.0 model selection tool based on AICc, and reconstruction performed by ML method, using all amino-acids in the alignment and 100 bootstraps. Tree was condensed based on bootstrap cutoff of 35.

Figure 3: Maximum likelihood based clustering of coral toxins. Sequence names are as following: coral sequence id, followed by evidence for expression (T stands for True and indicates protein was detected in proteomic analysis of nematocyst, while F stands for False and lack of detection). Last part of sequence name is assigned annotation based on Uniprot ToxProt toxins enriched by Anemone toxins. HMM-clustering generated groups are marked on the tree and groups not generated by HMM clustering, but detected by ML clustering are marked by *.

***Maximium Parsimony (MP) clustering:***

Figure 4: Maximum parsimony based clustering of coral toxins.
